# Altered Interoceptive Awareness in High Habitual Symptom Reporters and Patients With Somatoform Disorders

**DOI:** 10.3389/fpsyg.2020.01859

**Published:** 2020-08-07

**Authors:** Tabea Flasinski, Angelika Margarete Dierolf, Silke Rost, Annika P. C. Lutz, Ulrich Voderholzer, Stefan Koch, Michael Bach, Carina Asenstorfer, Eva Elisabeth Münch, Vera-Christina Mertens, Claus Vögele, André Schulz

**Affiliations:** ^1^Clinical Psychophysiology Laboratory, Institute for Health and Behaviour, University of Luxembourg, Esch-sur-Alzette, Luxembourg; ^2^Mental Health Research and Treatment Center, Faculty of Psychology, Ruhr-University Bochum, Bochum, Germany; ^3^Schön Klinik Roseneck, Prien am Chiemsee, Germany; ^4^Therapiezentrum Justuspark, Bad Hall, Austria; ^5^Ambulante Psychosoziale Rehabilitation Salzburg, Salzburg, Austria

**Keywords:** interoceptive awareness, medically unexplained symptoms, somatoform disorder, symptom reporting, interoception

## Abstract

**Objective:** Altered interoception may play a major role in the etiology of medically unexplained symptoms (MUS). It remains unclear, however, if these alterations concern noticing of signals or if they are limited to the interpretation of signals. We investigated whether individuals with MUS differ in interoceptive awareness as assessed with the Multidimensional Assessment of Interoceptive Awareness (MAIA) questionnaire.

**Methods:** Study 1: A total of 486 individuals completed the Screening for Somatoform Disorders (SOMS-2). Thirty-two individuals each of the upper and lower decile of the SOMS distribution (low symptom reporters/LSR, high symptom reporters/HSR) completed the MAIA. Study 2: MAIA scores of individuals diagnosed with somatoform disorder (SFD; *n* = 26) were compared to individuals with major depressive disorder (MDD; *n* = 25) and healthy controls (HC; *n* = 26).

**Results:** HSR had lower scores than LSR on the MAIA scales Not-Distracting and Not-Worrying. The SFD and MDD groups showed lower scores than HC on the MAIA scales Not-Distracting, Self-Regulation, and Trusting. The MDD group scored lower than the other two groups on the scales Body Listening and Attention Regulation. There were no group differences on the scale Noticing.

**Conclusion:** HSR, SFD, and MDD patients do not differ from HC in the awareness of noticing of interoceptive signal processing, whereas cognitive facets of interoception, such as distraction or self-regulation are differentially affected. This highlights the necessity of including specifically targeted interventions, which improve interoceptive awareness, in the prevention and treatment of SFDs.

## Introduction

At least one third of the general population experiences “medically unexplained symptoms” (MUS) once in a lifetime (Kroenke and Price, 1993). MUS are defined as symptoms that do not have or are not sufficiently explained by a medical cause and hence cannot be treated by conventional medical interventions. About 3 to 10% of the general population develop persistent MUS (Toft et al., 2005; Verhaak et al., 2006). Individuals experiencing MUS often report a decrease in their quality of life and impairments in daily functioning and frequently undergo unnecessary medical examinations and treatments (Barsky et al., 2005). MUS are still under-diagnosed in primary care (Kljakovic, 2009), even though it is assumed that 40 to 49% of primary care patients usually visit a general practitioner for at least one MUS (Haller et al., 2015). In addition, according to the Diagnostic and Statistical Manual of Mental Disorders, Fourth Edition (DSM-IV)^1^ (American Psychiatric Association [APA], 2000), MUS are a main diagnostic criterion of somatoform disorders (SFDs).

Even though they are associated with adverse consequences, the mechanisms behind the generation of MUS remain poorly understood. Alterations in interoception (i.e., the processing and perception of signals from within the body) (Farb et al., 2015; Khalsa et al., 2018) are presumed to play a major role in the development and maintenance of MUS (Rief and Broadbent, 2007). Several models of interoception are described in the literature. Despite important differences between these models, one commonality concerns the notion that interoception can be sub-divided into multiple facets, such as the passive noticing of interoceptive sensations, the allocation and regulation of attentional resources toward interoceptive sensations, or their interpretation (e.g., positive or negative evaluation) (Vaitl, 1996; Mehling et al., 2012; Herbert and Pollatos, 2019; Quadt et al., 2019). Current models of MUS make assumptions concerning alterations in some facets of interoception, whereas other facets remain discounted. The concept of somatosensory amplification (SSA) (Barsky et al., 1988; Barsky and Wyshak, 1990) posits that individuals with MUS show alterations in the interpretation of interoceptive sensations; that is, they have the tendency to experience “normal” interoceptive sensations as intense and disturbing. This model was recently extended to not include the amplification of interoceptive sensations but also external signals that a pose threat to oneself; therefore, it is nowadays referred to as somatic threat amplification (Köteles and Witthöft, 2017). Nevertheless, here we use the original conceptualization of SSA, as the present studies focus on interoceptive sensations. While we examined in the current study model assumptions of the SSA by investigating multiple facets of potentially altered interoception in MUS (including noticing and interpretation of interoceptive signals), we also included additional facets of interoception, which are not explicitly addressed by the SSA model.

Interoceptive measures are typically classified based on their methodology of assessment. For example, the correspondence between the occurrence of interoceptive sensations (e.g., caused by a heartbeat) and their perception as assessed through heartbeat perception tasks is defined as a measure of performance reflecting the facet “interoceptive accuracy” (IAc) (Garfinkel et al., 2015; Murphy et al., 2019). Although IAc is considered one of the most basic facets of interoception (Garfinkel et al., 2015; Forkmann et al., 2016), it may have one important shortcoming in that it reflects one facet of perception of interoceptive sensations, independent of their interpretation (Quadt et al., 2019). To assess multiple facets of interoception, alternative assessment approaches, such as self-reports, need to be integrated. There are various self-report measures of interoception, which reflect (meta-cognitive) beliefs about one’s IAc (i.e., confidence ratings in heartbeat perception tasks, Interoceptive Accuracy Scale/IAS: Murphy et al., 2019; Murphy et al., 2020) or about the tendency to focus one’s attention on interoceptive sensations (e.g., Body Perception Questionnaire/BPQ: Porges, 1993; Murphy et al., 2019). These models use a narrow definition of “metacognitive” interoceptive awareness (IAw) as the correspondence between beliefs and performance in interoceptive tasks (related to IAc or interoceptive attention) (Garfinkel et al., 2015; Murphy et al., 2019). A wider definition used in other models conceptualize self-report measures as indicators of different facets of IAw (Mehling et al., 2012, Mehling, 2016).

The majority of questionnaire measures (such as the IAS or the BPQ) assess single aspects of interoception, and are, therefore, not suitable for the investigation of potential alterations that may be limited to certain facets of interoception associated with MUS. For example, they do not distinguish between different attention styles (e.g., listening to bodily signals, directing one’s attention to elsewhere etc.) toward interoceptive signals, even though this is an important differentiation as these attention styles can be either seen as maladaptive and associated with somatization, or adaptive and enhancing resilience (Mehling, 2016). To overcome this shortcoming, the Multidimensional Assessment of Interoceptive Awareness (MAIA) (Mehling et al., 2012), a self-report IAw questionnaire, has been designed to differentiate between multiple facets of interoception. The eight MAIA subscales can be summarized into five partially independent factors of IAw. Although self-report questionnaires may have limitations in the investigation of perceptual processes, one could argue that they partially reflect the awareness of consecutive stages of interoceptive signal processing, ranging from the awareness of early sensory aspects (i.e., scale “Noticing”), mid-stages of automatic attentional and affective responses (“Not-Distracting,” “Not-Worrying”), and late stages, including cognitive processes, such as attention (“Attention Regulation”), the awareness of mind-body integration (“Emotional Awareness,” “Self-Regulation,” “Body Listening”) to “Trusting” interoceptive sensations (Mehling et al., 2012). Importantly, the MAIA scales allow for the differentiation between adaptive and maladaptive attention styles, which are assumed to be disturbed in MUS. In line with the theoretical assumptions underlying the MAIA, we interpret the MAIA scales as reflecting different facets of IAw.

The MAIA has previously been employed in patient samples with a variety of bodily symptoms. Individuals with past or current low back pain scored lower on all eight subscales, suggesting they are less often aware of their bodily sensations (Mehling et al., 2013). In another study, fibromyalgia patients showed lower scores on Not-Distracting and Trusting, and higher scores on Noticing (Valenzuela-Moguillansky et al., 2017), while yet in another study patients only scored lower on the Trusting subscale (Borg et al., 2018). These patterns of alterations in IAw may be specific for lower back pain and fibromyalgia patients. Furthermore, they do not differentiate between patients reporting pain with or without a medical explanation. Hence, it is yet unclear if medically unexplained pain conditions are accompanied by specific alterations in facets of IAw as assessed by the MAIA. The aim of the current study was, therefore, to address this yet unresolved question.

As a self-report measure, the MAIA is well-suited to differentiate between “Noticing” of interoceptive signals and their interpretation in MUS. According to the SSA model, MUS result from the “normal” noticing of benign interoceptive sensations, but an increased attentional focus on and negative affective responses (i.e., worries) to these signals (Barsky et al., 1988; Barsky and Wyshak, 1990; Rief and Broadbent, 2007). While these processes may be reflected by the scales Noticing, Not-Distracting, and Not-Worrying, the MAIA contributes to addressing additional questions. For example, it may help to clarify whether deficits in attention and emotion regulation play a role in the maintenance of the “vicious circle” of increased attentional and affective responses to interoceptive sensations and their occurrence. Although self-regulatory deficits have been proposed as a potentially maintaining factor of MUS (Brown, 2004; De Gucht and Maes, 2006), it is yet unknown if attention and emotion regulation associated with interoceptive sensations is altered in MUS. Based on the model underlying the MAIA, the facet “Noticing” does not differentiate between positive and negative affective biases, whereas MUS are characterized by negative affective biases, potentially indicated by higher scores on the Not-Worrying scale (Mehling et al., 2012, Mehling, 2016). In summary, and in line with the SSA model, for individuals with MUS, we would expect normal scores on the Noticing scale, but lower scores on the Not-Distracting and Not-Worrying scales. Furthermore, to the best of our knowledge, this is the first study to address the remaining facets of IAw, which may play a role in the maintenance of MUS.

In Study 1, we assessed individuals with multiple MUS (high symptom reporters/HSR), but without diagnosed SFD, as some of these individuals can be seen as high risk for developing SFD at a later stage (Rief and Hiller, 1999, 2003; Bogaerts et al., 2008, 2010b; Constantinou et al., 2013; Walentynowicz et al., 2015, 2017). In addition, any abnormalities at this stage could be of etiological importance and not just a result of the fully developed condition. We compared their MAIA scores to low symptom reporters (LSR), i.e., individuals with few or no MUS. In Study 2, we investigated differences in MAIA scores between SFD patients, patients with major depressive disorder (MDD), and healthy controls (HC). Individuals with MDD served as clinical control group, as MDD represents a typical comorbidity of SFD (Kroenke and Price, 1993; Henningsen et al., 2003; Rief and Barsky, 2005) and MDD may also be associated with impaired interoception (Dunn et al., 2007; Paulus and Stein, 2010; Terhaar et al., 2012; Furman et al., 2013). For both studies, we hypothesized lower IAw in the MUS than in the non-MUS groups (Study 1: HSR < LSR; Study 2: SFD < MDD = HC). More specifically, we expected that MUS groups show normal scores on the Noticing scale, but lower scores on the Not-Distracting and Not-Worrying scales. Furthermore, we tested for the assumption that MUS may be characterized with reduced scores in scales indicating self-regulation and interpretation associated with interoceptive sensations.

## Study 1

### Materials and Methods

#### Participants

Four-hundred eighty-six participants completed the SOMS-2 online, which was distributed via web-based advertisements, and social and public media in Luxembourg. The SOMS-2 (Screening für Somatoforme Störungen) (Rief et al., 1992; Rief and Hiller, 1999) assesses the number of MUS experienced within the past 2 years. Based on SOMS-2 scores, we identified respondents in the upper and lower centile of the distribution, i.e., HSR and LSR. While LSR reported less than six symptoms [*M* = 1.9; (*SD* = 1.8)], HSR reported between 20 and 50 symptoms [*M* = 27.4; (*SD* = 6.8)]. Potential participants of the upper and lower decile were screened with a telephone interview to check for the following exclusion criteria: (a) BMI <19 or >30 kg/m^2^, (b) acute or chronic illnesses, including mental disorders, (c) pregnancy, (d) proneness to faint, (e) current medication other than occasional non-opioid and non-steroidal pain killers (more than one third of all days in the past 2 weeks) or oral contraceptives, (f) regular alcohol (one 2 cl beverage per day on average or more) or other drug consumption, and (g) current treatment for MUS. Eligible participants (HSR, *n* = 32; LSR, *n* = 32) were invited to take part in the study. Average length of time between online screening and the lab assessment was 7.60 months (*SD* = 5.78). Participants provided written informed consent and received 20 € in gift vouchers for participation. The study was approved by the Ethics Review Panel of the University of Luxembourg.

#### Measures

##### Screening for somatoform disorders (SOMS)

We used an online adaptation of the SOMS-2 to screen participants; to validate the results of the online adaptation and to ensure that high/low MUS distress was still present at the day of lab assessment, the SOMS-7T was completed by participants on the lab assessment day (Screening für Somatoforme Störungen) (Rief and Hiller, 2003). Both questionnaires contain 53 symptoms, of which six items are covering gender-specific symptoms (five items specific for women, one item specific for men) (American Psychiatric Association [APA], 2000). For the SOMS-2, participants are asked to indicate on a yes/no-scale whether they have experienced any of the symptoms in the past 2 years. In the SOMS-7T, participants are asked to indicate on a scale from 0 (not at all) to 4 (very strong) whether they have experienced any of the symptoms in the past 7 days. For both questionnaires, respondents are asked to only indicate symptoms without a medical explanation. Internal consistencies have been shown to be very good (Screening für Somatoforme Störungen) (Rief et al., 1992).

##### Beck’s depression inventory II (BDI-II)

The BDI-II is a 21-item questionnaire to assess the severity of depression (Beck et al., 1996). Answers can be given on a scale from 0 (the symptom is not present) to 3 (the symptom is always present).

##### Multidimensional assessment of interoceptive awareness (MAIA)

The MAIA is a 32-item questionnaire that assesses IAw on a 6-point Likert scale from 0 (Never) to 5 (Always). For both studies, the German translation of the MAIA was used (Bornemann et al., 2015). For a full description of the subscales, see Mehling et al. (2012). The scales have adequate to excellent internal consistencies (Bornemann et al., 2015).

#### Procedure

Before participating in a larger study setup, participants completed the SOMS-7T, BDI-II, and MAIA while a researcher was present in the room.

#### Statistical Analyses

Spearman correlations were calculated across MAIA scales. Differences on the demographic variables were assessed with independent-samples *t* tests for continuous and chi-square tests for categorical variables. The differences on the MAIA subscales were assessed with one-way analyses of covariance (ANCOVA) with the BDI-II sum score and sex as covariates to control for depressive symptoms and sex differences between the groups, respectively. Assumptions of normal distribution (Shapiro-Wilk) and homogeneity of variances (Levene) were tested for all questionnaires. In case of violation, we verified the results of the parametric test using the Mann–Whitney *U* test (for all MAIA scales: after controlling for the variance of sex and BDI-II using standardized residuals in a multiple regression model). Cronbach’s alpha was assessed to evaluate internal consistency. Partial Eta squared was used as a measure of effect size. The analyses were conducted with SPSS 26, and a significance level of 0.05 was used for all tests.

### Results

#### Participant Characteristics

Overall, participants ranged in age from 18 to 57 years (HSR range 18–57 years; LSR 19–52 years). HSR and LSR did not differ in age, but there were significantly more women in the HSR than in the LSR group (Table 1).

**TABLE 1:** Participant characteristics for Study 1 (*N* = 64).

	**LSR (*n* = 32)**		**HSR (*n* = 32)**		***t*/χ2 test**		
	***M*/*n***	***SD***	***M*/*n***	***SD***	***t*/χ2**	***df***	***p***
Age (years)	26.59	7.07	28.66	9.45	0.99	62	0.33
Sex (f/m)	22/10		29/3		4.73	1	0.03
SOMS-2	1.84	1.73	27.38	6.83	20.51^a^	62	<0.001
SOMS-7T	3.28	3.82	13.53	6.62	7.58^b^	62	<0.001
BDI-II	4.72	4.50	15.16	9.44	5.65^c^	62	<0.001

*LSR, low symptom reporters; HSR, high symptom reporters; SOMS, Screening for Somatoform Disorders; BDI, Beck’s Depression Inventory; M, mean; n/N, sample size; SD, standard deviation; df, degrees of freedom. Alternative non-parametric analyses: ^*a*^*U* = 0.0; *Z* = −6.90; *p* < 0.001; ^*b*^*U* = 94.5; *Z* = −5.63; *p* < 0.001; ^*c*^*U* = 133.0; *Z* = −5.10; *p* < 0.001.*

#### Medically Unexplained Symptoms

Cronbach’s alpha was very good (SOMS-2 α = 0.93; SOMS-7T α = 0.91). For both scales, assumptions of normal distribution and homogeneity of variances were violated. LSR reported significantly less MUS than HSR in both scales (Table 1). There was a significant positive correlation between the SOMS-2 and SOMS-7T [*r*(62) = 0.74, *p* < 0.01]. This indicates that the number of symptoms over the last 2 years (trait) is associated with the symptoms over the last 7 days (state) and, furthermore, it validates the group assignment based on trait MUS scores.

#### Depressive Symptoms

Cronbach’s alpha for the BDI-II was very good (α = 0.93). Assumptions of normal distribution and homogeneity of variances were violated for the BDI-II. LSR showed lower BDI-II scores than HSR (Table 1).

#### Interoceptive Awareness

**FIGURE 1:**
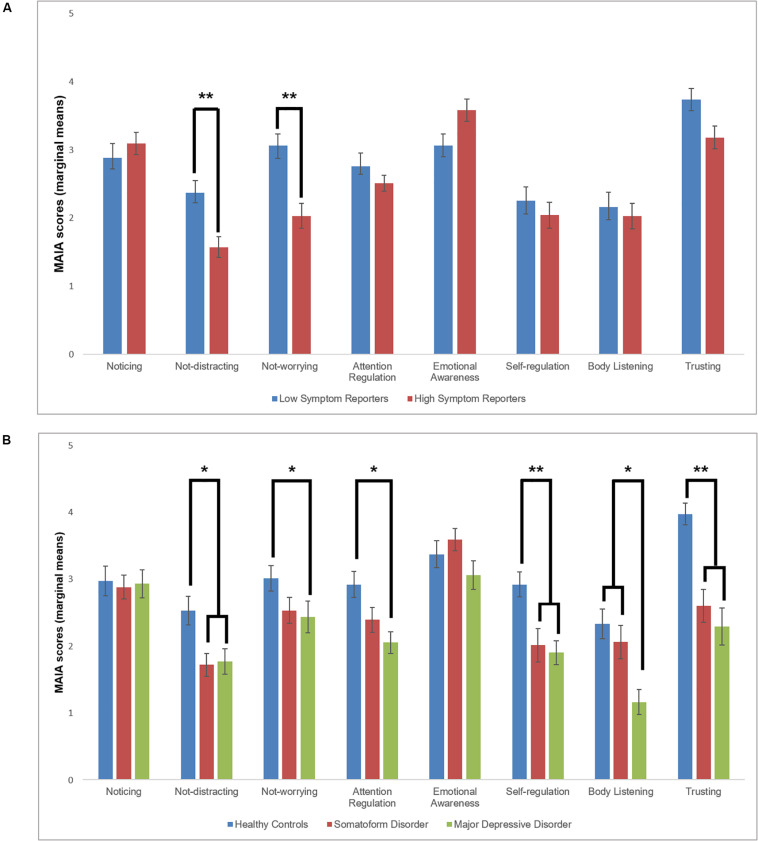
**(A)** Marginal means (after controlling for depressive symptoms and sex) of group differences in MAIA scales between high (*n* = 32) and low symptom reporters (*n* = 32; Study 1). **(B)** Marginal means (after controlling for age) of group differences in MAIA scales between patients with somatoform disorders (*n* = 26), major depressive disorder (*n* = 25), and healthy control individuals (*n* = 26; Study 2). Error bars represent one standard deviation. *p < 0.05; **p < 0.01 after correction for multiple comparisons.

Figure 1 summarizes descriptive statistics for the subscales of the MAIA. Cronbach’s alpha for the MAIA scales was good to excellent, ranging from 0.71 for Not-Distracting and Not-Worrying to 0.91 for Self-Regulation (Table 2). Assumption of normal distribution was violated for the scales Emotional Awareness, Self-Regulation, and Trusting, whereas the assumption of homogeneity of variances was violated for the scales Attention Regulation and Trusting.

**TABLE 2:** Spearman correlations among MAIA scales in the total sample of Study 1 (*N* = 64).

**Scale**	**1**	**2**	**3**	**4**	**5**	**6**	**7**	**α**
(1) Noticing	–							0.78
(2) Not-Distracting	–0.06	–						0.71
(3) Not-Worrying	−0.27*	0.19	–					0.71
(4) Attention Regulation	0.49**	–0.15	–0.01	–				0.85
(5) Emotional Awareness	0.59**	–0.20	−0.30*	0.38**	–			0.82
(6) Self-Regulation	0.33**	–0.08	0.04	0.66**	0.36**	–		0.91
(7) Body Listening	0.48**	0.07	0.05	0.63**	0.47**	0.71**	–	0.86
(8) Trusting	0.16	0.03	0.23	0.28*	0.20	0.35**	0.40**	0.82

*Correlations are significant at *p* < 0.05; **correlations are significant at *p* < 0.01.

Inter-correlations between MAIA scales are presented in Table 2. We observed multiple (mostly positive) correlations among the subscales of the MAIA, except for the scales Not-Distracting and Not-Worrying, which were uncorrelated with almost all other scales.

There were no significant group differences on the subscales Noticing [*F*(1,60) = 0.56, *p* = 0.55, η^2^ = 0.009], Attention Regulation [*F*(1,60) = 0.04, *p* = 0.85, η^2^ = 0.001], Emotional Awareness [*F*(1,60) = 2.97, *p* = 0.09, η^2^ = 0.05], Self-Regulation [*F*(1,60) = 2.38, *p* = 0.13, η^2^ = 0.04], Body Listening [*F*(1,60) = 2.10, *p* = 0.15, η^2^ = 0.03], and Trusting [*F*(1,60) = 1.72, *p* = 0.19, η^2^ = 0.03]. The HSR group showed significantly lower scores on the subscales Not-Distracting [*F*(1,60) = 6.86, *p* = 0.01, η^2^ = 0.10] and Not-Worrying [*F*(1,60) = 9.61, *p* = 0.003, η^2^ = 0.14] than the LSR group. The pattern of results remains almost identical when repeating the analysis of those scales with violated assumptions using the Mann–Whitney *U* Test (Attention Regulation: *U* = 508.5; *Z* = −0.47; *p* = 0.96; Emotional Awareness: *U* = 392.5; *Z* = −1.61; *p* = 0.11; Self-Regulation: *U* = 444.0; *Z* = −0.91; *p* = 0.36; Trusting: *U* = 443.0; *Z* = −0.93; *p* = 0.35).

## Study 2

### Methods

#### Participants

Participants were recruited from a rehabilitation outpatient hospital (Ambulante Psychosoziale Rehabilitation, Salzburg, Austria) and a psychosomatic hospital (Schön Klinik Roseneck, Prien am Chiemsee, Germany). To recruit participants in Luxembourg, we contacted psychotherapists (for patients only), distributed leaflets, and placed web-based advertisements (for patients and healthy participants). After phone screening, eligible participants were invited to take part in a Structured Clinical Interview for DSM-IV Axis I Disorders (SCID-I; Wittchen et al., 1997) to check for inclusion and exclusion criteria. Participants who met the DSM-IV (American Psychiatric Association [APA], 2000) criteria for SFD with MUS or MDD, and participants without a diagnosis of a mental disorder were eligible for participation. As the current study aims at investigating IAw in MUS, individuals with hypochondriasis, symptoms that are limited to somatosensory paresthesia (e.g., conversion disorder) or dysmorphophobia, were not eligible. Participants with MDD served as a clinical control group, as one of the most frequent comorbidities of SFD is MDD. Furthermore, we recruited healthy control (HC) participants.

Exclusion criteria were identical to those of Study 1. Furthermore, potential participants were excluded in case of (a) diagnosis of substance use disorder or dependency, and (b) the consumption of neuroleptic drugs or tricyclic anti-depressants. Other psychotropic drugs were allowed in the SFD and the MDD group only. Participants were also excluded if they reported any psychotic symptoms in the SCID-I.

All participants provided written informed consent. The study was approved by the Ethics Review Panel of the University of Luxembourg, and the ethics committees at the Ludwig Maximilian University Munich, Germany and the Federal State of Salzburg, Austria. Participants recruited in the psychosomatic hospital and those who were tested in Luxembourg received 60 € for participation, while participants from the rehabilitation outpatient hospital did not receive any compensation, as the ethics committee of the Federal State of Salzburg explicitly requested that participants would not receive any compensation, which would be viewed as an incentive to participate in the study; instead, participation should be completely voluntary.

#### Measures

##### Structured interview for DSM-IV axis I disorders (SCID-I)

The SCID-I is a semi-structured clinical interview for the assessment of the major DSM-IV Axis I diagnoses, with a duration of 30 to 120 min. The German version of the SCID-I was used for this study (Wittchen et al., 1997).

**Questionnaires (SOMS, BDI-II, MAIA)** were identical to Study 1.

#### Procedure

Diagnostic and Statistical Manual of Mental Disorders, Fourth Edition diagnoses were established using the SCID-I by A.M.D. or V.C.M. on a separate assessment day. Participants were asked to complete the questionnaires before participating in a larger study setup.

#### Statistical Analyses

Spearman correlations were calculated across MAIA scales. Group differences (SFD, MDD, and HC) on the demographic variables were assessed with one-way analyses of variances (ANOVA). To consider the age difference between the three groups, the differences on the MAIA subscales were assessed with analyses of covariances (ANCOVA) with age as covariate. Assumptions of normal distribution (Shapiro-Wilk) and homogeneity of variances (Levene) were tested for all questionnaires. In case of violation, we verified the results of the parametric test using the Kruskal–Wallis *H* test (for all MAIA scales: after controlling for the variance of age using standardized residuals in a multiple regression model). Bonferroni-corrected *t* tests for independent samples were used as *post hoc* test (Mann-Whitney *U* tests for non-parametric analyses). Cronbach’s alpha was assessed to evaluate internal consistency. Partial Eta-squared was used as a measure of effect size. A significance level of 0.05 was used for all tests and the analyses were conducted with SPSS 26.

### Results

#### Participant Characteristics

Overall, participants ranged in age from 18 to 64 years (SFD: 19–64 years; MDD: 18–59 years; HC: 19–52 years). Participants in the MDD and SFD group were significantly older than HC participants (Table 3). The sex distribution between the three groups did not differ significantly.

**TABLE 3:** Participant characteristics for Study 2 (*N* = 77).

	**HC (*n* = 26)**		**SFD (*n* = 26)**		**MDD (*n* = 25)**		***F*/χ2 test**		
	***M*/*n***	***SD***	***M*/*n***	***SD***	***M*/*n***	***SD***	***F*/χ2**	***df***	***P***
Age (years)	29.42	10.94	38.04	14.06	41.68	11.06	6.91	2,74	0.002
Sex (f/m)	18/8		21/5		13/12		4.86	2	0.09
SOMS-2	2.44	3.50	13.96	7.26	10.46	6.57	24.05^a^	2,74	<0.001
SOMS-7T	2.38	2.84	10.55	6.58	8.80	6.57	14.20^b^	2,74	<0.001
BDI-II	3.54	12.80	14.19	10.14	24.20	13.03	28.76^c^	2,74	<0.001

*HC, Healthy control; SFD, Somatoform Disorder; MDD, Major Depressive Disorder, SD, Standard deviation; SOMS, Screening for Somatoform Disorders; BDI, Beck’s Depression Inventory; df, degrees of freedom. Alternative non-parametric analyses (*df* = 2): ^*a*^*H* = 28.46; *p* < 0.001; ^*b*^*H* = 28.50; *p* < 0.001; ^*c*^*H* = 36.32; *p* < 0.001.*

In the SFD group (*n* = 26), 5 individuals received a somatization disorder diagnosis, 17 received a somatoform pain disorder, and 4 received a diagnosis of undifferentiated SFD according to DSM-IV-TR criteria. Comorbidities in the SFD group included the following: three dysthymia, three MDD, one depressive disorder not otherwise specified, one agoraphobia without history of panic disorder, three panic disorder with agoraphobia, three social anxiety disorder, one panic disorder, one generalized anxiety disorder, two anxiety disorder not otherwise specified, and four specific phobias. In the MDD group (*n* = 25), all participants were diagnosed with primary MDD. Current comorbidities in the MDD group were the following: one panic disorder with agoraphobia, three obsessive–compulsive disorder (two were in partial remission), one anxiety disorder not otherwise specified, four specific phobia, two social anxiety disorder, and two generalized anxiety disorder. One individual reported a past anorexia nervosa.

#### Medically Unexplained Symptoms

Cronbach’s alpha for both questionnaires was very good (SOMS-2 α = 0.90; SOMS-7T α = 0.89). For the SOMS-7T, both assumptions of normal distribution and homogeneity of variances were violated, whereas for the SOMS-2, only the normal distribution was not achieved. On both scales, the HC group reported a lower number of MUS than both clinical groups. Although the SFD group reported descriptively more MUS than the MDD group in the past 2 years and the past 7 days, both groups did not differ significantly. There was a significant positive correlation between the SOMS-2 and SOMS-7T [*r*(75) = 0.83, *p* < 0.01].

#### Depressive Symptoms

Cronbach’s alpha for the BDI-II was very good (α = 0.96). Assumptions of normal distribution and homogeneity of variances were violated for the BDI-II. The MDD group had higher BDI-II scores than the SFD and HC group, and the SFD group also exhibited higher BDI-II scores than the HC group.

#### Interoceptive Awareness

Figure 1B shows an overview of means and standard deviations for each subscale. Cronbach’s alpha for the MAIA subscales was acceptable to very good and ranged from 0.57 for Not-Worrying to 0.91 for Trusting (Table 3). Assumption of normal distribution was violated for the scales Body Listening and Trusting, whereas homogeneity of variance was not achieved for Trusting only.

Inter-correlations between MAIA scales are presented in Table 4. We found comparable patterns as observed in Study 1, with multiple positive correlations among the subscales of the MAIA, except for the scales Not-Distracting and Not-Worrying, which were uncorrelated with almost all other scales.

**TABLE 4:** Spearman correlations among MAIA scales in the total sample of Study 2 (*N* = 77).

**Scale**	**1**	**2**	**3**	**4**	**5**	**6**	**7**	**α**
(1) Noticing	–							0.70
(2) Not-Distracting	–0.09	–						0.70
(3) Not-Worrying	–0.06	0.03	–					0.57
(4) Attention Regulation	0.40**	0.03	0.26**	–				0.87
(5) Emotional Awareness	0.46**	0.04	0.00	0.36**	–			0.83
(6) Self-Regulation	0.16*	0.11	0.10	0.50**	0.29**	–		0.84
(7) Body Listening	0.43**	0.06	0.07	0.52**	0.44**	0.64**	–	0.88
(8) Trusting	0.12	0.21	0.36**	0.53**	0.18	0.53**	0.42**	0.91

*Correlations are significant at *p* < 0.05; **correlations are significant at *p* < 0.01.

There were no significant group differences in the mean scores for the subscales Noticing [*F*(2,73) = 0.03, *p* = 0.97, η^2^ = 0.001], and Emotional Awareness [*F*(2,73) = 1.98, *p* = 0.15, η^2^ = 0.05]. The SFD and MDD group had lower mean values than the HC group on Self-Regulation [*F*(2,73) = 7.99, *p* = 0.001, η^2^ = 0.18] and Trusting [*F*(2,73) = 13.91, *p* < 0.001, η^2^ = 0.28; all *post hoc* tests: *p*s < 0.01]. The MDD group had lower mean scores than the HC group on Not-Worrying [*F*(2,73) = 4.47, *p* = 0.02, η^2^ = 0.11] and Attention Regulation [*F*(2,73) = 4.01, *p* = 0.02, η^2^ = 0.10], but the SFD group did not differ from the MDD and HC group on these scales. The SFD group showed higher scores on Not-Distracting than the HC group [*F*(2,73) = 3.81, *p* = 0.03, η^2^ = 0.10; *post hoc* test: *p* < 0.05], whereas the MDD group did not differ from the two remaining groups significantly. On Body Listening, the MDD group had significant lower mean values than the SFD and HC group [*F*(2,73) = 7.53, *p* = 0.001, η^2^ = 0.17; all *post hoc* tests: *p*s < 0.05], but the SFD and HC group did not differ significantly from each other. The pattern of results remains almost identical when repeating the analysis of those scales with violated assumptions using the Kruskal–Wallis *H* test (Emotional Awareness: *H* = 4.45; *p* = 0.11; Body Listening: *H* = 9.56; *p* = 0.008; SFD vs. MDD: *p* = 0.09; SFD vs. HC: *p* = 1; MDD vs. HC: *p* = 0.009; Trusting: *H* = 17.74; *p* < 0.001; SFD vs. MDD: *p* = 1; SFD vs. HC: *p* = 0.001; MDD vs. HC: *p* < 0.001).

## Discussion

The present studies are the first to investigate the different facets of IAw as assessed by the MAIA in two independent samples with HSR and fully manifested SFD. The aim was to clarify which facets of interoception are potentially altered in individuals with MUS. In line with existing theories (Barsky and Wyshak, 1990; Rief and Broadbent, 2007; Köteles and Witthöft, 2017), we expected that MUS is associated with increased attentional focus on interoceptive sensations and negative affective responses (i.e., worries) to these sensations, indicated by lower scores on the Not-Distracting and Not-Worrying scales (Mehling et al., 2012). Furthermore, as the scale “Noticing” does not differentiate between positive and negative affective responses to interoceptive sensations (Mehling, 2016), with the latter being indicative of MUS, we expected this scale not to be altered in MUS. In accordance with our hypothesis, individuals experiencing MUS showed lower IAw on some facets that are assessed with the MAIA. More specifically, in Study 1, HSR worried more about experiences of pain and discomfort (Not-Worrying). In addition, they had the tendency to distract or ignore sensations of pain and discomfort (Not-Distracting). In contrast, HSR did not notice more physical sensations than LSR (Noticing). In Study 2, individuals diagnosed with a SFD experienced their body as less safe and trustworthy than HC (Trusting). Furthermore, they had the tendency to focus their attention on sensations of pain or discomfort (Not-Distracting) and had difficulties in the ability to regulate distress by attention to body sensations (Self-Regulation). Again, there were no differences between SFD and HC in noticing of physical sensations (Noticing).

The MAIA aims to differentiate between multiple facets of IAw (Mehling et al., 2012): The Noticing scale is thought to indicate the awareness of passive, non-evaluative registration of interoceptive signals. Not-Distracting and Not-Worrying are assumed to reflect the awareness of attentional and affective responses to interoceptive sensations. Attention Regulation, Emotional Awareness, Self-Regulation, and Body Listening mirror the capacity to regulate attentional and affective responses, whereas Trusting represents the interpretation of interoceptive sensations. Based on the rationale underlying its construction (Mehling et al., 2012), these scales may represent self-report measures of subsequent stages of interoceptive signal processing: Noticing is assumed to reflect early, sensory-perceptual processes of signal processing, while Not-Distracting and Not-Worrying assess mid-stages of automatic attentional and affective responses. The remaining facets might be indicative of late, self-regulatory, and interpretative stages. This view is supported by studies demonstrating that mindfulness and exercise interventions increase IAw as assessed with the scales Attention Regulation, Emotional Awareness, Self-Regulation, Body Listening, and Trusting. In contrast, scales reflecting early and mid-stages, which are conceptualized as partially automatic facets of IAw (Noticing, Not-Distracting, Not-Worrying), remain unaffected by these interventions (Bornemann et al., 2015; Mehling et al., 2017). As retrospective self-reports may have limitations for the investigation of (pre-conscious) perceptual processes, however, future studies should include behavioral and psychophysiological indicators of interoception to clarify if MUS are associated with the selective alteration of mid-stages of interoceptive signal processing. As MUS are characterized by attentional and interpretative biases toward interoceptive sensations (Barsky and Wyshak, 1990; Rief and Broadbent, 2007), it has been hypothesized that individuals experiencing MUS selectively report lower IAw scores on the scales reflecting these facets (e.g., Not-Distracting, Not-Worrying, Trusting), but not on the Noticing scale, because this scale does not differentiate between interoceptive sensations of positive or negative affective valence (Mehling, 2016).

First, and in line with this hypothesis, there were no differences in “Noticing” of interoceptive sensations between groups in either study, i.e., between HSR and LSR, or between SFD and HC individuals. This suggests that the passive, non-evaluative registration of interoceptive signals is unaffected by MUS. It remains to be clarified in future studies if individuals with and without MUS show alterations in the amplitude of interoceptive sensations or if just the awareness of these interoceptive sensations is comparable between individuals with and without MUS. This would require a psychophysiological assessment of afferent bodily signals. If the comparable scores of Noticing between individuals with and without MUS were due to a normal amplitude of interoceptive sensations, this would be in disagreement with the perception-filter model, according to which increased interoceptive sensations are the first stage of symptom perception (Rief and Barsky, 2005; Rief and Broadbent, 2007). Instead, our findings are in line with central assumptions of the SSA model (Barsky et al., 1988; Barsky and Wyshak, 1990), which imply that not passive noticing of interoceptive sensations is altered in individuals with MUS, but subsequent attentional and affective responses. This view is also supported by previous studies demonstrating that Not-Worrying is negatively correlated with self-reports on the Somatosensory Amplification Scale, whereas Noticing is not (Borg et al., 2018). Second, lower scores on the Not-Distracting subscale in individuals with MUS in both study samples suggest attentional bias toward bodily sensations, which has previously been reported in studies using both self-report and behavioral assessments of attentional processes (Pennebaker, 1982; Bantick et al., 2002; Houtveen et al., 2003). Third, lower scores on the subscales Trusting (Study 2), Not-Worrying (Study 1), and Self-Regulation (Study 2) imply that individuals with MUS interpret physical sensations as more threatening and have less regulatory resources to adequately cope with these emotions (Hitchcock and Mathews, 1992; Schwarz et al., 2017). These alterations may contribute to the vicious circle of increased attention, fear and anxiety, and symptom perception as proposed by the SSA model (Barsky and Wyshak, 1990).

In general, the findings from both studies are partially in line with findings of previous studies administering the MAIA to individuals with physical symptoms (Mehling et al., 2013; Valenzuela-Moguillansky et al., 2017). Individuals with current or past low back pain show lower IAw on all eight subscales (Mehling et al., 2013), while in our study, individuals with MUS only scored lower on some subscales when compared to individuals with few or no MUS. It has to be acknowledged, however, that the sample size of this study was higher (*n* = 435) (Mehling et al., 2013), which allows for smaller effects to become significant. Moreover, sample characteristics between the two studies were different. While Mehling et al. (2013) tested individuals with past or current low back pain (with and without a medical explanation), the present sample included individuals with a high number of (heterogeneous) MUS (Study 1) or a fully manifested SFD or MDD (Study 2). These sample differences (lower statistical power, higher current symptom severity, and no medical explanation) may have contributed to the different patterns of alterations in IAw scales. These differences may also explain the scores on the scales Not-Distracting, Self-Regulation, Body Listening, and Trusting scores of HSR/SFD patients in the current studies, which were even lower than in the study by Mehling et al. (2013). In a study with fibromyalgia patients of a comparable sample size (*n* = 60) (Valenzuela-Moguillansky et al., 2017), patients scored lower on Not-Distracting and Trusting, which is in line with our findings on HSR and SFD patients. In contrast to our findings, however, Valenzuela-Moguillansky et al. (2017) also reported higher scores on the subscale Noticing. This may constitute a fundamental difference between SFD and fibromyalgia and suggests that fibromyalgia is not only associated with attentional bias toward interoceptive sensations, such as pain (McDermid et al., 1996; Peters et al., 2000; Crombez et al., 2004), but also with altered awareness of passive, non-evaluative noticing of interoceptive sensations (Ceko et al., 2012; Sluka and Clauw, 2016). Treatments for MUS should be designed to selectively target those IAw dimensions, which are altered in the respective sample.

The current results suggest that different facets of IAw are involved in the development and maintenance of MUS. High risk for SFDs has been conceptualized as a high number of self-reported physical symptoms (41), often operationalized as the number of MUS (Rief and Hiller, 1999, 2003). One approach to address etiological factors underlying SFD may be the investigation of this high-risk population (i.e., HSR), as any abnormalities at this stage could be of etiological importance, and not just a result of the fully developed condition. Individuals at high risk for developing a SFD, as well as SFD and MDD patients, do not differ from HC in terms of awareness of passive, non-evaluative noticing of interoceptive sensations. It seems that cognitive facets of interoception, such as distraction or self-regulation, are differentially affected. While the high-risk population still worry about experiences of pain and discomfort (Not-Worrying), patients with a clinical diagnosis do not seem to worry more than HC but they have difficulties in regulating distress by attention to body sensations (Self-Regulation). One explanation for this finding could be “blunting” as a coping strategy by patients with SFD, as concerned individuals with SFD have experienced symptoms for a much longer period of time than HSR individuals. An alternative explanation may lie in the fact that all SFD patients were in treatment for their symptoms, which might cause them to worry less and feel less emotional distress, as they have found an explanation for their symptoms.

Although reduced Self-Regulation may be a feature of progressing SFD, it has to be acknowledged that this scale did not differentiate between SFD and MDD patients, which suggests that other facets of IAw have to be considered. MDD patients scored lower on the Attention Regulation scale than HC. This in line with previous studies showing deficits in multiple facets of the attentional system in MDD (Hammar and Årdal, 2009), which may be due to a dysregulation of the central noradrenergic system (Itoi and Sugimoto, 2010). Importantly, Body Listening differentiates between MDD and SFD in our sample, which suggests that this pattern represents a specific feature of IAw in MDD. It may be speculated that due to a profound dysfunction of regulatory systems in the body [e.g., vagal dysfunction (Koschke et al., 2009) and HPA axis dysregulation (Varghese and Brown, 2001)], MDD could be seen as the end result of losing the connection to one’s own body (Paulus and Stein, 2010). Nevertheless, in Study 1 depressiveness does not seem to play a role for the group differences in IAw, as the latter remain significant after controlling for depression scores. In summary, despite some overlaps in the patterns of IAw in SFD and MDD, the present multi-faceted approach illustrates that the MAIA may serve as an additional diagnostic instrument to assess specific alterations in IAw in mental disorders associated with physical symptoms.

When considering IAc based on behavioral tasks (e.g., heartbeat perception tasks), there is an inconsistency in the literature as to whether SFD patients show normal (Mussgay et al., 1999; Schaefer et al., 2012) or decreased IAc (Bogaerts et al., 2008, 2010b; Pollatos et al., 2011; Weiss et al., 2014). As our studies showed that only attentional and interpretational facets of IAw are altered in SFD, we would argue that lower IAc (correspondence between reported and actual interoceptive sensations) in SFD is the result of a distortion of reported interoceptive sensations (e.g., distorted by an attentional bias) (Bogaerts et al., 2010a; Walentynowicz et al., 2018), whereas interoceptive sensations may be identical to healthy individuals. Literature on the relationship of MAIA scores and IAc in SFD is scarce. Two studies investigating both parameters of interoception in fibromyalgia patients and healthy control individuals show inconsistent patterns of either no relationship (Borg et al., 2018) or positive correlations of IAc with the scales Attention Regulation, Emotional Awareness, and Body Listening (Valenzuela-Moguillansky et al., 2017). As scores on these scales do not seem to differ between SFD patients and healthy controls in neither of both earlier studies, and fibromyalgia patients and control individuals did not differ in IAc anyway, this would suggest that the (additional) assessment of IAw with the MAIA is necessary to assess the specific alterations of interoceptive signal processing in MUS.

## Limitations

Although the diagnostic interviews clearly differentiated between SFD and MDD individuals, the SOMS scores only exhibited descriptively higher numbers of experienced symptoms in the SFD than the MDD group. Individuals in the SFD group were mainly diagnosed with a somatoform pain disorder; therefore, our results may not generalize to individuals with somatic symptoms unrelated to pain. Comorbid mental disorders in the SFD and MDD groups might have contributed to the current findings. Furthermore, individuals of the three groups in Study 2 differed in age. Age possibly has an effect on interoception, as it seems that IAc declines with higher age (Khalsa et al., 2009). As this effect is not yet established for IAw assessed with the MAIA, possible age-related effects on IAw should be addressed in future studies. Moreover, there was a sex difference between the groups in Study 1. Even though there is a higher prevalence of MUS in women (Osugo et al., 2017), a possible sex effect on IAw should be addressed by future studies (Grabauskaitë et al., 2017). Nevertheless, we controlled for both sex and age effects in our statistical models by introducing these variables as covariates to account for these differences. It cannot be ruled out, however, that significant group differences or the large female/male ratio may have masked effect, which the inclusion of covariates cannot fully control for. Moreover, it cannot be ruled out that the numerous inter-correlations between MAIA scales may have partially masked or contributed to these findings. To investigate the differential contribution of MUS and depressive symptoms, future studies should investigate both characteristics in a large sample reflecting their entire range (and not extreme groups), which allows for a regression analytical approach. The current study is solely based on self-report data. As individuals with MUS tend to report stronger maladaptive responses to interoceptive sensations if they are framed as symptoms (which is the case for the Not-Distracting and Not-Worrying scale) (Van Den Bergh and Walentynowicz, 2016), the findings of our studies might be partially explained by this bias. This would, however, not explain differences in the Trusting scale, whose items exclusively have a positive connotation. In addition to self-reports, future studies should include behavioral (e.g., IAc) and physiological indicators (e.g., heartbeat-evoked potentials) to reveal if alterations in interoceptive facets in MUS/SFD are limited to meta-cognitive beliefs on the awareness of interoceptive signals, or whether these findings also translate into alterations of mid-stages of interoceptive signal processing. These methods should be complemented with MAIA scales to validate the current findings. Finally, although carefully selected, the sample sizes of both studies were comparatively small. Notwithstanding, our findings with regard to internal consistency and scale inter-correlations were comparable or somewhat higher than previously reported (Mehling et al., 2013; Bornemann et al., 2015; Cali et al., 2015; Borg et al., 2018), suggesting the reliable assessment of IAw in the present samples.

## Conclusion

In summary, the results of the present two independent studies suggest that IAw is lower in individuals who experience MUS with regard to Not-distracting, Not-worrying, Self-regulation, and Trusting. At the same time, Noticing of bodily signals is unaffected by MUS. This is in line with assumptions of the SSA model (Barsky et al., 1988; Barsky and Wyshak, 1990). Mindfulness might decrease the discrepancy between expected and perceived interoceptive signals (i.e., “prediction error”) (Farb et al., 2015). As our findings imply that only attentional and interpretative facets of interoception are altered, mindfulness-based interventions (Van Ravesteijn et al., 2013) may help to reduce this prediction error in MUS by focusing on actual interoceptive sensations (which might be unchanged), thereby supporting more appropriate expectations about interoceptive signals. Furthermore, cognitive reappraisal focused on the re-framing of interoceptive signals with positive emotions could be another promising treatment approach.

## Data Availability Statement

The raw data supporting the conclusions of this article will be made available by the authors, without undue reservation.

## Ethics Statement

The studies involving human participants were reviewed and approved by the Ethics Review Panel of the University of Luxembourg, the Ethics Committee at the Ludwig Maximilian University Munich, Germany and the Ethics Committee of the State of Salzburg, Austria. The patients/participants provided their written informed consent to participate in this study.

## Author Contributions

AS and CV conceived study design and acquired funding. AD, SR, TF, UV, SK, MB, CA, EM, and V-CM assessed the data. TF, AD, SR, and AS analyzed the data. TF and AS wrote the first manuscript draft. All authors edited and approved manuscript.

## Conflict of Interest

The authors declare that the research was conducted in the absence of any commercial or financial relationships that could be construed as a potential conflict of interest.

## Footnotes

1 As the SCID-I was not available for DSM-V at the time of data collection, DSM-IV diagnostic criteria are used in the present studies.
